# Results of scoping review do not support mild traumatic brain injury being associated with a high incidence of chronic cognitive impairment: Commentary on McInnes et al. 2017

**DOI:** 10.1371/journal.pone.0218997

**Published:** 2019-09-16

**Authors:** Grant L. Iverson, Justin E. Karr, Andrew J. Gardner, Noah D. Silverberg, Douglas P. Terry

**Affiliations:** 1 Department of Physical Medicine and Rehabilitation, Harvard Medical School, Boston, Massachusetts, United States of America; 2 Spaulding Rehabilitation Hospital and Spaulding Research Institute, Charlestown, Massachusetts, United States of America; 3 MassGeneral Hospital *for* Children^™^ Sports Concussion Program, Boston, Massachusetts, United States of America; 4 Home Base, A Red Sox Foundation and Massachusetts General Hospital Program, Charlestown, Massachusetts, United States of America; 5 Department of Psychology, University of Victoria, Victoria, British Columbia, Canada; 6 Hunter New England Local Health District Sports Concussion Program and Centre for Stroke and Brain Injury, School of Medicine and Public Health, University of Newcastle, Newcastle, New South Wales, Australia; 7 Division of Physical Medicine & Rehabilitation, University of British Columbia, Vancouver, British Columbia, Canada; 8 Rehabilitation Research Program, Vancouver Coastal Health Research Institute, Vancouver, British Columbia, Canada; Nathan S Kline Institute, UNITED STATES

## Abstract

A recently published review of 45 studies concluded that approximately half of individuals who sustain a single mild traumatic brain injury (MTBI) experience long-term cognitive impairment (McInnes et al. Mild Traumatic Brain Injury (mTBI) and chronic cognitive impairment: A scoping review. *PLoS ONE* 2017;12:e0174847). Stratified by age, they reported that 50% of children and 58% of adults showed some form of cognitive impairment. We contend that the McInnes et al. review used a definition of “cognitive impairment” that was idiosyncratic, not applicable to individual patients or subjects, inconsistent with how cognitive impairment is defined in clinical practice and research, and resulted in a large number of false positive cases of cognitive impairment. For example, if a study reported a statistically significant difference on a single cognitive test, the authors concluded that *every* subject with a MTBI in that study was cognitively impaired–an approach that cannot be justified statistically or psychometrically. The authors concluded that impairment was present in various cognitive domains, such as attention, memory, and executive functioning, but they did not analyze or report the results from any of these specific cognitive domains. Moreover, their analyses and conclusions regarding many published studies contradicted the interpretations provided by the original authors of those studies. We re-reviewed all 45 studies and extracted the main conclusions from each. We conclude that a single MTBI is not associated with a high incidence of chronic cognitive impairment.

## Introduction

A recently published scoping review of the literature concluded that approximately half of individuals who sustain a single mild traumatic brain injury (MTBI) experience long-term cognitive impairment [1]. The authors identified 45 studies that met their inclusion criteria. Through their synthesis and analysis, they reported that “1963 participants out of 3593, or approximately 55% of our sample collapsed across all time points showed cognitive impairment” (page 10). Stratified by age, they reported that 50% of children and 58% of adults showed some form of cognitive impairment (page 11). They asserted that “a large proportion of individuals with a single mTBI will continue to demonstrate measurable impairment in various cognitive domains including executive function, learning/memory, attention, processing speed, and language function long after the initial injury” (page 13). They stated that the published literature to date represents a “gross underestimation” (pages 13 and 14) of the extent of cognitive impairment caused by a single MTBI, and “it is possible that our results represent a further underestimation of the incidence of persistent cognitive impairment following a single mTBI” (page 14).

We disagree with the findings and conclusions summarized above from the *scoping review* published by McInnes and colleagues [1]. Their conclusions are fundamentally different from, or run counter to, findings from numerous meta-analyses of the MTBI neuropsychological literature [2–15]. A scoping review is relatively new and still evolving approach to knowledge synthesis. Standardized methodology and reporting guidelines are not yet available [16, 17], but there is consensus that the main purpose of scoping reviews is to examine the extent and nature of available research in a defined subject area [16–18]. A scoping review can be helpful for a subject area that has not previously been comprehensively reviewed, often to determine if there is sufficient evidence to conduct a systematic review [18, 19]. This seems to be the reason why McInnes et al. selected this method of knowledge synthesis (i.e., they wrote: “the studies that assess long-term cognitive outcomes in singly-concussed individuals have not been gathered and reviewed”; page 2).

There are essential differences between scoping reviews and systematic reviews. Scoping reviews do not have rigid exclusion criteria and do not formally evaluate the quality of evidence [16, 19], consistent with their goal of summarizing the breadth of literature. In contrast, systematic reviewers perform both of these tasks in order to reduce bias in trying to answer specific research questions, such as prognosis or treatment efficacy. Whereas systematic reviews often include a meta-analysis of aggregated quantitative data, scoping reviews generally provide only a descriptive narrative [16]. Scoping reviews may also include a “descriptive numerical summary” to map the time, location, and source of available research, typically reported as a frequency count of studies with certain characteristics [17–20].

McInnes et al. did not exclude studies with a high risk of bias and did not perform quality appraisals of included studies, consistent with scoping review methodology [19]. However, they went well beyond numerically summarizing the number and type studies available. They recoded and synthesized quantitative information, and from these analyses, drew conclusions about the incidence of long-term cognitive impairment following MTBI. This falls outside the purview of a scoping review and exposed McInnes et al. to the risk of flawed conclusions. In their original description of scoping review, Arksey and O’Malley explained that “unlike a systematic review the scoping study does not seek to ‘synthesize’ evidence or to aggregate findings from different studies… because the scoping study does not seek to assess quality of evidence and consequently cannot determine whether particular studies provide robust or generalizable findings”[18]. McInnes et al. used systematic review techniques to synthesize evidence without an assessment of the risk for bias or consideration of how bias might influence their results. A systematic review that omits these elements provides “critically low” confidence in their conclusions and “should not be relied on to provide an accurate and comprehensive summary of the available studies” ([21, page 6].

We have three primary concerns regarding the methodology used to synthesize and summarize data in their scoping review. First, their definition of “cognitive impairment” was idiosyncratic, not applicable to individual patients or subjects, and inconsistent with how cognitive impairment is defined in clinical practice and research. Their definition resulted in a large number of false positive cases of “cognitive impairment.” In the McInnes et al. review, participants in the original studies were dichotomized into “cognitively impaired” and “cognitively unimpaired” groups. McInnes et al. [1] defined individuals as having cognitive impairment “if their outcome measure score significantly differed from those of the control groups or the normative data, or if they were below author-identified cut-off scores” (page 7). Of the 45 original studies, only 9 studies (20%) were dichotomized based on author-identified definitions of cognitive impairment [22–30]. The remaining studies did not define “cognitive impairment” in their text and were dichotomized by McInnes et al. based on differences on significance testing between the MTBI and control groups. Two original studies that defined cognitive impairment in an *a priori* manner did not report the incidence of cognitive impairment in their sample, and those studies were classified by McInnes et al. based on group comparisons [31, 32]. If a study reported a statistically significant difference on a single cognitive test, the authors concluded that *every subject in that study with a MTBI was cognitively impaired*. This method represents a misunderstanding or misapplication of statistical significance testing. A statistically significant difference between an MTBI group and a control group means that the difference between the means of groups is not likely to be zero, thus the associated term in statistical testing is *null hypothesis testing*. A p-value does not provide us information regarding the practical or clinical significance of the difference, the magnitude of the difference, or whether the difference is large enough to classify people into one group or another. A statistically significant difference between groups on a test or tests cannot be used to accurately or reliably classify individual subjects as cognitively impaired. Classifying *every* subject in the MTBI group as cognitively impaired in these instances also does not make sense from a practical standpoint. There are likely several individuals in the MTBI groups who performed better than the mean of the control groups and/or whose scores would be interpreted as broadly normal (e.g., average or better) based on using traditional neuropsychological interpretation schemes. These methods artificially inflate the percentage of individuals classified as cognitively impaired. Second, the authors concluded that impairment was present in various cognitive domains, such as attention, memory, and executive functioning (page 13 of the Discussion), but they did not analyze or report the results from any of these cognitive domains in their review. Third, their analyses and conclusions regarding many published studies contradicted the interpretations provided by the original authors of the studies (e.g., [33–35]).

## Materials and methods

### Review of 45 articles relating to cognitive functioning following a single MTBI

We re-reviewed the 45 articles identified in the McInnes et al. scoping review [1] to examine the sampling strategy and statistical techniques used when determining if participants who experienced an MTBI had cognitive impairment. Further, we thought it would be useful to provide a summary statement for each of these 45 studies based on the data and original authors’ conclusions. We did not seek to complete our own scoping review, systematic review, or meta-analysis of these studies. Prior systematic reviews and meta-analyses have examined this topic in great detail [2–15].

From each article, we extracted the percentage of the MTBI sample with a complicated MTBI (i.e., macrostructural trauma-related intracranial abnormalities visible on computed tomography or magnetic resonance imaging), as well as the sample size, age (mean and standard deviation), and recruitment settings for the MTBI and control groups. We also extracted the number of group comparisons for cognitive outcomes (i.e., the number of test scores that were analyzed/compared between the MTBI and control groups) and the number of statistically significant group differences. We determined whether the original authors classified individual subjects as cognitively impaired or not, and whether the original authors drew conclusions about whether or not subjects were cognitively impaired. We examined whether other factors that may influence cognitive functioning were reported in the original studies (i.e., whether the original study assessed for pre-morbid or current intellectual functioning, or mental health problems). This does not necessarily mean that these variables were used in statistical models to control for their potential effect when assessing for cognitive differences between groups. Three authors (JK, AG, and DT) with experience conducting systematic reviews [11, 12, 36, 37] completed extractions for all of the articles. Each study was reviewed by two authors. We provided a brief summary of the statistical findings and implications of each article, using quotations from the original articles whenever possible.

## Results

The findings were consolidated into Table 1. Several studies summarized in the McInnes et al. scoping review [1] did not include means, SDs, or effect sizes for the statistical comparisons between groups on cognitive testing (e.g., [22, 27, 38–42]). As such, it is not possible to draw conclusions from those studies regarding the magnitude of the difference between the MTBI group and the control group. Moreover, for most of the studies it is not known whether a subgroup within the MTBI group met criteria for cognitive impairment. As noted above, the scoping review by McInnes and colleagues [1] came to fundamentally different conclusions in comparison to numerous published meta-analyses of the MTBI neuropsychological literature [2–15]. McInnes and colleagues [1] identified some more recently published studies, since 2013, that were not included in previous systematic reviews and meta-analyses because they were published after those searches were performed [22, 23, 34, 43–50]. However, those more recently published studies, as a rule, did not compute the percentages of the MTBI sample that met criteria for cognitive impairment, nor did they yield results suggestive of chronic cognitive impairment (see Table 1).

**Table 1:** Summary of studies 3, 6, 12, and >12 months post injury.

Summary of Studies 3 Months Post Injury
Rieger et al., 2013 [23]*; Country: USA; % Comp MTBI = 0%; MTBI N = 39, Age = 13.7 (2.9), Presented to emergency department; Control N = 30, Age = 13.2 (2.0), Trauma controls. IQ = Yes; Psych. = No Summary: On the ImPACT, children with mTBI performed significantly worse than the trauma controls on the Visual Memory composite at three months. Comparisons = 4, Differences = 1. Definition of CI: One ImPACT composite score ≤30^th^ percentile (of either Verbal Memory, Processing Speed, and Reaction Time). % CI: 96% in MTBI group, 85% control group
Phillipou et al., 2013 [34]*; Country: Australia; % Comp MTBI = 0%; MTBI N = 26, Age = 13.1 (2.3), Emergency departments (but not admitted or imaged); Control N = 29, Age = 12.2 (2.2), Community advertisements or siblings. IQ = Yes; Psych. = No Summary: MTBI group had fewer correct responses on ImPACT immediate/delayed word memory and delayed design memory subtests. Traditional ImPACT domain scores were not examined. Comparisons = 9, Differences = 3. Definition of CI: NR. % CI: NR
Tay et al., 2010 [38]*; Country: Singapore; % Comp MTBI = 0%; MTBI N = 38, Age = 40.6 (14.7), Mild head injury clinic; Control N = 38, Age = 39.0 (15.9), Community participants recruited via advertisements and word of mouth. IQ = No; Psych. = Yes Summary: N = 31 at follow-up. Results section reports that there are no group differences in the dimensions of prospective memory or the overall number of errors, but that the MTBI group makes more prospective memory errors. Comparisons = 15, Differences = 1. Definition of CI: NR. % CI: NR
Kwok et al., 2008 [51]*; Country: China; % Comp MTBI = 100; MTBI N = 15, Age = 39.1 (11.5), Emergency Department; Control N = 19, Age = 44.5 (7.5), Community convenience sampling. IQ = Yes; Psych. = Yes Summary: Repeated measures ANOVAs were conducted for 11 cognitive outcomes. The time x group interaction was significant for 3 outcomes. There was a significant between-group difference between the mTBI and control groups for 1 outcome at 3 months post-injury based on post-hoc comparisons. Comparisons = 3, Differences = 1. Definition of CI: NR. % CI: NR
Su et al., 2014 [22]**; Country: China; % Comp MTBI = 0%; MTBI N = 213, Age = 39.8 (SEM = 0.7), Consecutive patients treated across 4 hospitals; Control N = NA, Age = NA. IQ = No; Psych. = Yes Summary: “Persistent cognitive impairment, most prominently on the subdomains of attention and delayed recall, were reported in 54/213 (25%) cases.” (p.113) Comparisons = 0, Differences = 0; Definition of CI: MoCA score < 25 points; % CI: 25%
Siman et al., 2013 [43]*; Country: USA; % Comp MTBI = 0%; MTBI N = 17, Age = 20.5 (5.8) for all participants admitted to emergency departments; Control N = 8, Age = 20.5 (5.8) for all participants, uninjured controls (details not specified). IQ = No; Psych. = No Summary: The authors reported the MTBI group performed worse on a processing speed and a working memory task at three months post injury. Scores/test statistics not presented in original study. Comparisons = 2, Differences = 2. Definition of CI: NR. % CI: NR
Ponsford et al., 2011 [52]*; Country: Australia; % Comp MTBI = 0%; MTBI N = 123, Age = 35.0 (13.1), Consecutive Emergency Department patients; Control N = 100, Age = 35.4 (10.7), Trauma Controls. IQ = No; Psych. = Yes Summary: ImPACT Visual Memory Composite was lower in MTBI group at 3-months post injury. Comparisons = 4, Differences = 1. Definition of CI: NR. % CI: NR
Pare et al., 2009 [53]*; Country: Canada; % Comp MTBI = NR; MTBI N = 37, Age = 26.7 (10.3), Emergency Department; Control N = 79, Age = 30.6 (11.9), Community recruitment. IQ = No; Psych. = Yes Summary: Control group had more education. On a novel dual-task paradigm assessing divided attention, MTBI participants had slower reaction times. The control participants made more errors. Comparisons = 10, Differences = 5. Definition of CI: NR. % CI: NR
Kinsella et al., 2014 [44]*; Country: Australia; % Comp MTBI = 28%; MTBI N = 50, Age = 76.5 (7.6), Hospital trauma services; Control N = 123/58, Age = 75.8/73.8, Orthopedic injury controls from trauma services and non-injured controls from community clubs/societies Summary: Participants with mTBI performed significantly worse on tests of prospective memory and set-shifting in comparison to non-injured controls, but not orthopedic injury controls. IQ = No; Psych. = No Comparisons = 6, Differences = 3. Definition of CI: NR. % CI: NR
Marsh & Smith (1995) [54]*; Country: New Zealand; % Comp MTBI = 0%; MTBI N = 15, Age = 27.07 (12.60), Consecutive patients treated in the hospital for concussion; Control N = 15, Age = 26.7 (11.6), Siblings, relatives, or companions of the MTBI group. IQ = Yes; Psych. = Yes Summary: Group differences in attentional and language functioning (PASAT, Stroop, COWAT). Comparisons = 8, Differences = 3. Definition of CI: NR. % CI: NR
de Boussard et al., 2005 [24]**; Country: Sweden; % Comp MTBI = 6.2%; MTBI N = 97, Age = 37.2, range: 15–65, Emergency Departments; Control N = 35, Age = 39, Local Advertisements. IQ = No; Psych. = No Summary: Comparing cognitive performances to control participants or normative samples, the researchers categorized a minority of participants as showing signs of cognitive impairment potentially due to MTBI at 3 months post-injury. Comparisons/Differences: NR. Definition of CI: For a brief computerized test battery, “if patients performed 1 SD worse than mean for the controls in at least 2 separate…tests, at least 2 occasions, they were coded as having signs of cognitive impairment, possibly due to MTBI.” (p. 54). For an extended neuropsychological test battery, “The performance was considered abnormal if 2 or more test results were below 1 SD or if the results of separate tests differed by 2 SD or more.” (p. 55). % CI: 8% with CI per computerized testing; 30% with CI per extended neuropsychological test battery
Hanten et al., 2013 [45]*; Country: USA; % Comp MTBI = 32.2%; MTBI N = 59; Age = Divided based on post-concussion symptoms: Low = 18.6 (5.3), High = 17.9 (4.0), Emergency Department; Control N = 27/58, Age = Non-injured Controls: 20.3 (5.1), Recruitment: NR; Age = Orthopedic Injury: 20.2 (5.5), Emergency Department. IQ = No; Psych. = No Summary: No difference between participants with mTBI or orthopedic injury on a test of updating working memory, but non-injured controls performed significantly better than both injured groups. Comparisons/Differences: NR. Definition of CI: NR. % CI: NR
Heitger et al., 2006 [55]*; Country: New Zealand; % Comp MTBI = 0%; MTBI N = 37, Age = 29.1 (12.7), Recruitment = Hospital; Control N = 37, Age = 29.2 (12.6), Psychology Department. IQ = Yes (as outcome); Psych. = No Summary: The researchers compared groups on an undefined number of scores derived from six tests. They identified 5 CVLT scores that were lower for the MTBI group. Comparisons/Differences: NR. Definition of CI: NR. % CI: NR
Bohnen et al., 1993 [30]**; Country: Netherlands; % Comp MTBI = 0%; MTBI N = 46, Age = 28.3 (14.9), Seeking medical attention; Control N = 43, Age = 29.2 (14.2), Pool of healthy volunteers. IQ = No; Psych. = Yes. Summary: Participants with MTBI and 3 or more post-concussion symptoms were significantly worse on the Stroop Color Word Interference test compared to participants with MTBI and 2 or fewer symptoms at 3 months post injury. Using the control participants as normative data, 7/10 participants with 3 or more symptoms at 3 months were <10^th^ percentile on the Stroop, and 1/36 participants with less than 3 symptoms at 3 months. Comparisons = 3, Differences = 2. Definition of CI: Using control participants scores, <10^th^ percentile on the Stroop: 17
Rotarescu & Ciurea (2008) [39]*; Country: Romania; % Comp MTBI = NR; MTBI N = 96, Age = 10.5 (3.4), Emergency department, hospitalized for MTBI; Control N = NA, Age = NA. IQ = Yes (as outcome); Psych. = Yes Summary: This study did not have a control group to compare neuropsychological scores to. It did not establish thresholds to classify patients as impaired. Comparisons = 0, Differences = 0. Definition of CI: NR. % CI: NR
Ponsford et al., 1999 [56]˟; Country: Australia; % Comp MTBI = 0%; MTBI N = 72/58, Age = 11.2/11.4 (2.7/3.2), Emergency Department; Control N = 49/47, Age = 10.9/12/3 (2.5/2.1), Trauma Controls. IQ = Yes; Psych. = Yes Summary: “There were no statistically significant differences in the performances of the mild THI and control groups on any neuropsychological measures at 3 months postinjury (Table 4).” (p.368) “By 3 months postinjury, symptoms had generally resolved, and there was no evidence of cognitive impairment.” (p. 370) Comparisons = 9, Differences = 0. Definition of CI: NR. Note: 17% of the MTBI group were noted to have mood/behaviors problems at 3 months post injury. When compared to the MTBI patients without problems, there were no significant neuropsychological differences. % CI: NR
Ponsford et al., 2000 [57]˟; Country: Australia; % Comp MTBI = 0%; MTBI N = 84, Age = 26.4 (13.9), Consecutive emergency department presentations; Control N = 53, Age = 30.7 (14.4), Trauma controls. IQ = Yes; Psych. = Yes Summary: “…results of univariate analyses indicated no significant differences between the groups on any of the neuropsychological measures at 3 months post-injury.” (p. 572) Comparisons = 6, Differences = 0; Definition of CI: NR; % CI: NR
Maillard-Wermelinger et al., (2009) [58]˟; Country: USA; % Comp MTBI = 18%; MTBI N = 186, Age = 12.0 (2.2), Emergency Department visits; Control N = 99, Age = 11.8 (2.2), Trauma Controls. IQ = Yes; Psych. = No Summary: “On the CANTAB, the groups did not differ on the Stockings of Cambridge and the mild TBI group unexpectedly performed better than the OI group on Spatial Working Memory.” (p. 330) “Children with mild TBI show limited evidence of deficits in executive functions, either cognitively or behaviourally, irrespective of injury characteristics.” (p. 330) Comparisons = 2, Differences = 0. Definition of CI: NR. % CI: NR
Levin et al., (1996) [33]˟; Country: USA; % Comp MTBI = NR; MTBI N = 36, Age = 10.1 (3.01), Prospective cohort who were hospitalized acutely for injury; Control N = 104, Age = 10.4 (3.2), Recruited from Community. IQ = Yes (as outcome); Psych. = No Summary: “To summarize this cross-sectional analysis, there was no evidence of a semantic or episodic memory deficit in the children who sustained a mild CHI as compared to a normal control group.” (p. 466) Comparisons = 11, Differences = 0. Definition of CI: NR. % CI: NR
Xu et al., (2014) [59]**, Country: China; % Comp MTBI = 0%; MTBI N = 118, Age = 39.3 (13.1), Prospective cohort recruited from hospital neurosurgery department within 5 days of injury; Control N = NA, Age = NA. IQ = No; Psych. = No. Summary: They concluded that post-acute plasma ghrelin levels were associated with “cognitive deterioration” at 3-months following injury. Comparisons = NR, Differences = NR. Definition of CI: Authors reported “Forty patients (33.9%) had cognitive deterioration three months after MTBI.” Authors did not provide an operational definition of what “cognitive deterioration” is, nor did they provide any cognitive tests scores. % CI: 33.9
**Summary of Studies 6 Months Post Injury**
Phillipou et al., 2013 [34]*; Country: Australia; % Comp MTBI: 0; MTBI N = 26, Age = 13.1 (2.3), Emergency departments (but not admitted or imaged); Control N = 29, Age = 12.2 (2.3), Community advertisements or siblings. IQ = Yes; Psych. = No Summary: MTBI group had slower reaction time on ImPACT design memory subtest. Traditional ImPACT domain scores were not examined. Comparisons = 9, Differences = 1. Definition of CI: NR. % CI = NR
Wong et al., 2010 [25]*; Country: Australia; % Comp MTBI: 0; MTBI N = 4, Age = 52 (17.9), Setting: NR; Control N = 10, Age = 48 (15.1), Community recruitment. IQ = No; Psych. = No Summary: “No statistically significant differences between the MTBI and control participants across subtests of general language, high-level language, and cognitive variables were revealed.” (p. 1160). Then, low scores were analyzed. 4/4 patients had at least one low score, out of 42 possible scores. Comparisons = 41, Differences = 0. Definition of CI: Each score that was -2 SD below the mean of the control group was deemed “anomalous.” % CI = 100% (4 out of 4 participants) had at least 1 low score (number of low scores, out of 42: 1, 2, 6, 13)
Ellemberg et al., 2007 [60]*; Country: Canada; % Comp MTBI: NR; MTBI N = 10, Age = 22.7, Female University Soccer Team; Control N = 12, Age = 22.3, Female University Soccer Team. IQ = No; Psych. = No Summary: A series of t-tests compared 19 scores derived from 10 tests identifying some significant differences between groups. Comparisons = 19, Differences = 6. Definition of CI: NR. % CI = NR
Miles et al., 2008 [26]**; Country: USA; % Comp MTBI: 0; MTBI N = 17, Age = 33.4, range 18–58, Consecutive patients with MTBI in the hospital; Control N = 29, Age = 35, range 18–61, Healthy Volunteers. IQ = No; Psych. = No Summary: Only 12 MTBI patients returned to the 6-month follow-up. 33% continued to experience cognitive impairment at 6 months post injury. Comparisons = 0, Differences = 0. Definition of CI: Scoring at or below the 5th percentile (-1.6 SD below the normative mean) on two or more cognitive tests. % CI = 33
Wrightson et al., 1995 [41]*; Country: New Zealand; % Comp MTBI: NR; MTBI N = 69, Age = 3.4, Hospital Accident Department; Control N = 77, Age = 3.4, Hospital Accident Department, Orthopedic Controls. IQ = No; Psych. = No Summary: A series of ANOVAs were conducted for 11 or more cognitive scores. The researchers found lower scores in the head injured sample on a single score at 6 months post injury, although the groups did not differ on this same score within a month of injury. Comparisons/Differences = NR. Definition of CI: NR. % CI = NR
Heitger et al., 2006 [55]*; Country: New Zealand; % Comp MTBI: 0; MTBI N = 37, Age = 29.1 (12.7), Hospital; Control N = 37, Age = 29.2 (12.6), Psychology Department. IQ = Yes (as outcome); Psych. = No Summary: The researchers compared groups on an undefined number of scores derived from 6 tests. They identified 4 CVLT scores that were lower for the MTBI group. Comparisons/Differences = NR. Definition of CI: NR. % CI = NR
Bohnen et al., 1993 [30]**; Country: Netherlands; % Comp MTBI: 0; MTBI N = 46, Age = 28.3 (14.9), Seeking medical attention; Control N = 43, Age = 29.2 (14.2), Pool of healthy volunteers. IQ = No; Psych. = Yes. Summary: Participants with MTBI and 3 or more post-concussion symptoms were significantly worse on the Stroop Color Word Interference test compared to participants with MTBI and 2 or fewer symptoms at 6 months post injury. Using the control participants as normative data, 6/9 participants with 3 or more symptoms at 6 months were <10^th^ percentile on the Stroop, and 1/37 participants with less than 3 symptoms at 6 months. Comparisons = 3, Differences = 2. Definition of CI: NR. % CI = Using control participants scores, <10^th^ percentile on the Stroop: 15
Babikian et al., 2011, 2013 [46, 61]**; Country: USA; % Comp MTBI: NR; MTBI N = 94, Age = 11.9 (2.5), Emergency Departments; Control N = 96/101, Age = OI controls: 12.8 (2.5), Emergency Departments, Non-injury controls: 12.2 (2.5), Schools. IQ = No; Psych. = Yes Summary: A series of mixed model analyses evaluated time x group interactions and tested main effects for group. These main effects showed significant differences, but had cognitive performances collapsed across all post-injury epochs (i.e., 1 month, 6 months, and 12 months). In turn, it was not possible to determine if there was unique impairment in cognitive functioning for any outcome at 6 months. Although criteria for CI were described for 12 months post injury, they were not described for 6 months post injury. Comparisons/Differences = NR. Definition of CI: NR. % CI = NR
Rotarescu & Ciurea (2008) [39]*; Country: Romania; % Comp MTBI: NR; MTBI N = 96, Age = 10.5 (3.4), Emergency department, hospitalized for MTBI. IQ = Yes (as outcome); Psych. = Yes Summary: This study did not have a control group to compare neuropsychological scores to. It did not establish thresholds to classify patients as impaired. Comparisons = 0, Differences = 0. Definition of CI: NR. % CI = NR
Muller et al., 2008 [27]**; Country: Norway; % Comp MTBI: 26; MTBI N = 59, Age = 35.1, range 18–74, Consecutive patients referred to dept. of neurosurgery; this study did not have a control group. IQ = No; Psych. = Yes (but not reported) Summary: 55 patients at 6-month follow-up. Minimal comment about cognitive impairment at 6 months, other than patients got better over time. Comparisons = 0, Differences = 0. Definition of CI: Performance on each test was defined as impaired if the score was 1.5 SDs below the normative data. A score < 35 in 2 of 6 domains was considered to indicate neuropsychological impairment. % CI = 34.5 (19/55)
Barrow et al., 2006 [35]˟; Country: USA; % Comp MTBI: NR; MTBI N = 9, Age = 41, Setting = NR; Control N = 9, Age = 39, Setting = NR. IQ = No; Psych. = No Summary: No significant group differences observed at 6 months post injury Comparisons = 2, Differences = 0. Definition of CI: NR. % CI = NR
**Summary of Studies 12 Months Post Injury**
Catale et al., 2009 [62]*; Country: Belgium; % Comp MTBI: NR; MTBI N = 15, Age = 8.31 (1.25), Hospital Admissions; Control N = 15, Age = 8.36 (1.22), Primary education schools. IQ = Yes; Psych. = Yes Summary: The researchers compared 14 scores from 7 tests of IQ, attention, and executive function, identifying 4 significant differences between groups (*p* < .05) and one marginally significant difference (*p* = .07). Comparisons = 14, Differences = 4. Definition of CI: NR. % CI = NR
Lee et al., 2008 [63]*; Country: USA; % Comp MTBI: 75%; MTBI N = 28, Age = 30.3 (8.6), Emergency Department; Control N = 18, Age = 34.3 (8.9), Setting: NR. IQ = No; Psych. = No Summary: At 12 months post-injury, mTBI and control participants differed significantly on CVLT-II Total Recall Trials 1–5 and Long Delay Free Recall. Comparisons = 5, Differences = 2. Definition of CI: NR. % CI = NR
Polissar et al., 1994 [64]*; Country: USA; % Comp MTBI: 50% of those scanned; MTBI N = 53, Age = 9.4 (NR), Emergency Department; Control N = 53, Age = 9.7 (SD = NR), Chosen from the classroom of the injured child (or a similar classroom). IQ = Yes (as outcome); Psych. = No Summary: “At 1 year post-injury, using either the Bonferroni or Schweder and Spjotvoll adjustments with all of the neurobehavioural variables, only Coding was significantly associated with case-control status (p<0.01).” Authors discuss how different approaches to multiple comparisons may lead to different findings. Comparisons = 33, Differences = 1. Definition of CI: NR. % CI = NR
Kashluba et al., 2008 [31]*; Country: USA; % Comp MTBI: 100%; MTBI N = 102, Age = 48.6 (16.4), Emergency Department; this study did not have a control group. IQ = No; Psych. = No Summary: The researchers interpreted the MTBI sample to have presented with mild to mild-moderate cognitive impairment on 7/10 cognitive tests. Comparisons/Differences = NR. Definition of CI: “By comparing each group’s mean score on the neuropsychologic measures versus each measure’s demographically appropriate normative data, a level of impairment for each neuropsychologic variable was determined. To do so, a prototypical patient was created based on the mean demographic characteristics of each group (i.e., a 49-year-old man with 12 years of education in the complicated mild TBI group” (p.908). % CI = NR
Romero et al., 2015 [47]*; Country: Canada; % Comp MTBI: 0; MTBI N = 49, Age = 30.86 (12.42), Emergency Department; this study did not have a control group. IQ = Yes; Psych. = Yes Summary: This study examined the association between SPECT perfusion and cognitive measures. Cognitive test scores were not presented or interpreted. Comparisons = 0, Differences = 0. Definition of CI: NR. % CI = NR
Stålnacke et al., 2007 [28]*; Country: Sweden; % Comp MTBI: NR; MTBI N = 16, Age = NR, Admitted to hospital; Control N = 16, Age = NR, Health controls chosen by the authors via personal contacts. IQ = No; Psych. = Yes Summary: McInnes et al. reported that 69 patients with MTBIs completed testing at one year post injury. However, only 16 of the 69 patients underwent testing at 1-year post injury. The MTBI group had significantly lower scores on 2 tests out of 23 comparisons (i.e., oral processing speed and psychomotor speed). Comparisons = 23, Differences = 2. Definition of CI: One score (out of 23 possible scores) that is -1.5 SDs below the normative mean. % CI = 69% of MTBI patients had one or more low scores, compared to 44% of controls (χ^2^ test, *p* = 0.025).
Chadwick et al., 1981 [65]*; Country: England; % Comp MTBI: NR; MTBI N = 29, Age Range = 5–14, Hospital Admissions, All MTBI patients had PTA of 1 hour to 7 days (thus those with moderate TBIs were included); Control N = 28, Age Range = 5–14, Orthopedic Controls. IQ = Yes (as outcome); Psych. = No. Summary: MTBI group worse than controls on manual dexterity, impulsivity, processing speed, and verbal intellect. “The mild head injury group had a mean level of cognitive functioning below the control group, but lack of any recovery during the follow-up period indicated that the intellectual impairment was not a consequence of the injury.” (p. 49) Comparisons = 19, Differences = 4. Definition of CI: NR. % CI = NR
Wrightson et al., 1995 [41]*; Country: New Zealand; % Comp MTBI: NR; MTBI N = 57, Age = 3.38, Hospital Accident Department; Control N = 77, Age = 3.40, Hospital Accident Department, Orthopedic Controls. IQ = No; Psych. = No Summary: A series of ANOVAs were conducted for 11 or more cognitive scores. The researchers found lower scores by the head injured sample on a single score at 12 months post-injury, although the groups did not differ on this same score within a month of injury. Comparisons/Differences = NR. Definition of CI: NR. % CI = NR
Heitger et al., 2006 [55]*; Country: New Zealand; % Comp MTBI: 0; MTBI N = 31, Age = 29.1 (12.7), Hospital; Control N = 31, Age = 29.2 (12.6), Psychology Department. IQ = Yes (as outcome); Psych. = No Summary: The researchers compared groups on an undefined number of scores derived from six tests. They identified no differences at 12 months. Comparisons/Differences = NR. Definition of CI: NR. % CI = NR
Anderson et al., 2001 [40]*; Country: Australia; % Comp MTBI: 0; MTBI N = 17, Age = 5.1 (1.5), Admitted to neurosurgery ward; Control N = 35, Age = 5.1 (1.9), Child care centers, kindergartens, and schools. IQ = Yes (as outcome); Psych. = Yes Summary: A series of repeated-measures ANOVAs evaluated group (i.e., mTBI vs. control) and time main effects and interactions with 14 different cognitive scores as dependent variables, identifying a single significant difference at 12 months post-injury for the Story Recall Test. The Story Recall Test was significantly different between groups at all time points. Comparisons/Differences = NR. Definition of CI: NR. % CI = NR
Babikian et al., 2011, 2013 [46, 61]**; Country: USA; % Comp MTBI: NR; MTBI N = 106, Age = 11.9 (2.5), Emergency Departments; Control N = 102/109, Age = OI controls: 12.8 (2.5), Emergency Departments, Non-injury controls: 12.2 (2.5), Schools. IQ = No; Psych. = Yes Summary: The researchers determined criteria for determining cognitive impairment at 12 months and conducted a follow-up study that predicted the presence of cognitive impairment among participants with head or non-head injuries. They found that type of injury did not predict the presence of cognitive impairment. Comparisons/Differences = NR. Definition of CI: “To identify a subset of subjects that show lingering neurocognitive problems by 12 months post-injury (for the mild TBI and other injury groups) relative to the non-injured control group, the proportion of the sample in each of the three groups that scored 1.5 or greater standard deviations below the mean of the non-injured controls on at least three or at least 4 of the 10 neurocognitive measures was calculated.” (p.889). % CI = 29%
Rotarescu & Ciurea (2008) [39]*; Country: Romania; % Comp MTBI: NR; MTBI N = 96, Age = 10.46 (3.35), Emergency department, hospitalized for MTBI. IQ = Yes (as outcome); Psych. = Yes Summary: This study did not have a control group to compare neuropsychological scores to. It did not establish thresholds to classify patients as impaired. Comparisons = 0, Differences = 0. Definition of CI: NR. % CI = NR
Waljas et al., 2015 [48]˟; Country: Finland; % Comp MTBI: 13.5; MTBI N = 126, Age = 37.8 (13.5), Consecutive emergency department patients; Control N = 36, Age = 36.9 (13.6), Community controls. IQ = No; Psych. = Yes Summary: N = 103 at follow-up. No significant differences at 1 year post-injury. Also, there were no significant differences when comparing cognition in complicated MTBI vs. uncomplicated MTBI. Comparisons = 1, Differences = 0. Definition of CI: NR. % CI = NR
Dikmen et al., 2001 [66]˟; Country: USA; % Comp MTBI: 20%, 4% requiring surgical intervention. % Mod-Sev TBI: Some subjects had prior head injury, more severe brain injuries. 48% with PTA > 24hr; MTBI N = 157, Age = 28.4 (11.4), Hospitalized cases with evidence of TBI; Control N = 109, Age = 31.1 (13.8), Trauma controls. IQ = Yes (as outcome); Psych. = No Summary: No significant differences at 1 year post-injury. Comparisons = 4, Differences = 0. Definition of CI: NR. % CI = NR
Zhou et al., 2013 [49]˟; Country: USA; % Comp MTBI: NR; MTBI N = 19, Age = 34 (11.5), Emergency Department; Control N = 12, Age = 35.1 (11.3), Setting: NR. IQ = No; Psych. = Yes Summary: No significant differences between mTBI and control participants on scores deriving from 6 different neuropsychological tests. Comparisons/Differences = NR. Definition of CI: NR. % CI = NR
Croall et al., 2014 [50]˟; Country: United Kingdom; % Comp MTBI: NR; % Moderate-Severe TBI: 21.7; MTBI N = 23, Age = Mild TBI: 39.1 (15.8), Moderate TBI: 40.0 (17.3), Emergency Department; Control N = 33, Age = 40.9 (15.3), Recruitment setting: NR. IQ = Yes; Psych. = No Summary: No group difference between TBI and control participants based on a single test of verbal fluency. Comparisons = 1, Differences = 0. Definition of CI: NR. % CI = NR
Maillard-Wermelinger et al., (2009) [58]˟; Country: USA; % Comp MTBI: 18; MTBI N = 186, Age = 12.0 (2.2), Emergency Department visits; Control N = 99, Age = 11.8 (2.2), Trauma Controls. IQ = Yes; Psych. = No Summary: “On the CANTAB, the groups did not differ on the Stockings of Cambridge and the mild TBI group unexpectedly performed better than the OI group on Spatial Working Memory.” (page 330). “Children with mild TBI show limited evidence of deficits in executive functions, either cognitively or behaviourally, irrespective of injury characteristics.” (page 330) Comparisons = 2, Differences = 0. Definition of CI: NR. % CI = NR
Jaffe et al., 1995 [32]˟; Country: USA; % Comp MTBI: NR; MTBI N = 40, Age = 6–15, Setting: NR; Control N = 40, Age = 6–15, Setting: NR. IQ = Yes (as outcome); Psych. = No Summary: Children with mTBI showed minimal to no differences in cognitive functioning compared to controls at 1 year post injury. Comparisons/Differences = NR. Definition of CI: “Although it is subjective, many would agree that the clinical significance of a difference of one-quarter of a standard deviation between a study group and its controls would be considered minor; one-half of a standard deviation, moderate; and one standard deviation, substantial” (p.18). % CI = NR
Levin et al., (1996) [33]˟; Country: USA; % Comp MTBI: NR; MTBI N = 36, Age = 10.1 (3.0), Prospective cohort who were hospitalized acutely for injury; Control N = 104, Age = 10.4 (3.2), Recruited from Community. IQ = Yes (as outcome); Psych. = No Summary: The MTBI group was not compared to the control group at 12 months post injury Comparisons = NR, Differences = NR; Definition of CI: NR. % CI = NR
**Summary of studies >12 Months Post Injury**
Mangels et al., (2002) [67]*; Country: Canada; % Comp MTBI: 10; MTBI N = 11, Age = 29.4 (3.3), Prospective hospital admissions; Control N = 10, Age = 32.3 (3.0), Family and friends of patients. IQ = Yes; Psych. = No Summary: Some tests were completed at 1.5 years post injury, others were completed at 3.6 years post injury. There were no differences between MTBI and control groups on traditional neuropsychological tests. Differences evident on a novel cognitive paradigm. “Overall, MTBI patients were impaired only when items were encoded under divided attention, indicating memory deficits that were secondary to deficits in the executive control.” (pg 2369) Comparisons = 28, Differences = 2. Definition of CI: NR. % CI = NR
Chadwick et al., 1981 [65]*; Country: England; % Comp MTBI: NR; MTBI N = 29, Age Range = 5–14, Hospital Admissions, those with PTA for 1 hour to 7 days (thus, people with moderate TBIs were included); Control N = 28, Age Range = 5–14, Orthopedic Controls. IQ = Yes (as outcome); Psych. = No Summary: “… data suggested that intellectual functioning in the mild head injury group was *not* affected by the injury.” (pg. 54). " …children with mild head injuries tended to have a level of intellectual performance below general population norms, the lack of any recovery phase indicated that their cognitive limitations almost certainly antedated their accident and were not in any way caused by brain injury." (pg. 60) Comparisons = 6, Differences = 1. Definition of CI: Persistent Intellectual Impairment defied as 1) having an increase in 24 points or more between injury and 1 year, 2) score at 2.25 years pot injury -1 SD below controls. % CI = 0
Anderson et al., 2001 [40]*; Country: Australia; % Comp MTBI: 0; MTBI N = 17, Age = 5.1 (1.5), Admitted to neurosurgery ward; Control N = 35, Age = 5.1 (1.9), Child care centers, kindergartens, and schools. IQ = Yes (as outcome); Psych. = No Summary: A series of repeated-measures ANOVAs evaluated group (i.e., mTBI vs. control) and time main effects and interactions with 14 different cognitive scores as dependent variables, identifying a significant difference at 30 months post-injury for only 2 scores: a Verbal Fluency Test and a Story Recall Test. However, the Verbal Fluency Test only showed a group difference at 30 months and not at any earlier testing occasions. Further, the Story Recall Test was significantly different between groups at all time points. Comparisons/Differences = NR. Definition of CI: NR. % CI = NR
Wrightson et al., 1995 [41]*; Country: New Zealand; % Comp MTBI: NR; MTBI N = 47, Age = 6.5, Hospital Accident Department; Control N = 52, Age = 6.5, Hospital Accident Department, Orthopedic Controls. IQ = No; Psych. = No Summary: A series of ANOVAs were conducted for 11 or more cognitive scores. The researchers found lower scores by the head injured sample on a single score at about 2–4 years post-injury, although the groups did not differ on this same score within a month of injury. Comparisons/Differences = NR. Definition of CI: NR. % CI = NR
McCauley & Levin (2004) [42]*; Country: USA; % Comp MTBI: Yes (but % NR); MTBI N = 17, Age = 15.3 (2.1), Prospective hospital admissions; Control N = 15, Age = 15.1 (2.5), Community recruitment for orthopedic controls. IQ = No; Psych. = No Summary: Group differences in prospective memory as measured by behavioral and reaction time data. Comparisons = 7, Differences = 4. Definition of CI: NR. % CI = NR
Geary et al., 2010 [68]*; Country: USA; % Comp MTBI: NR; MTBI N = 40, Age = 34.5 (10.2), Community advertisements; Control N = 35, Age = 32.5 (10.8), Community advertisements. IQ = Yes; Psych. = Yes Summary: Participants with and without MTBI were compared on 11 scores from the CVLT-II, finding a significant difference on only Trial 1 of the list-learning condition. Comparisons = 11, Differences = 1. Definition of CI: NR. % CI = NR
Konrad et al., 2010 [29]**; Country: Germany; % Comp MTBI: 0; MTBI N = 33, Age = 36.7 (12.4), Based on patient records; Control N = 33, Age = 37.0 (12.0), Community advertisements. IQ = No; Psych. = Yes Summary: Significant differences were observed based on MANOVAs for all cognitive domains, with many individual tests showing univariate group differences. Comparisons = 21, Differences = 15. Definition of CI: “Performance…at least 1.5 Standard Deviations below the mean of the controls in two or more cognitive domains” (p.1203). % CI = 42.4%
Vanderploeg et al., (2005) [69]*; Country: USA; % Comp MTBI: NR; MTBI N = 254, Age = 38.4 (2.51), Veterans assessed ~16 years post discharge; Control Group #1: Normal Controls (Veterans without Motor Vehicle Accidents or an MTBI), N = 3,057, Age = 38.2 (2.5); Control Group #2 (Veterans with Motor Vehicle Accidents without MTBI), N = 521, Age = 38.4 (2.5). IQ = Yes; Psych. = Yes Summary: No statistically significant differences on traditional neuropsychological scores. When examining more nontraditional indices, the MTBI group had a lower percentage of participants who continued to PASAT trial 3 and a larger proactive inference effect on a list learning task compared to controls. Comparisons = 20, Differences = 2. Definition of CI: NR. % CI = NR
Jaffe et al., 1995 [32]˟; Country: USA; % Comp MTBI: NR; MTBI N = 40, Age = 6–15, Setting: NR; Control N = 40, Age = 6–15, Setting: NR. IQ = Yes (as outcome); Psych. = No Summary: Children with mTBI showed minimal to no differences in cognitive functioning compared to controls at 3 years post injury. Comparisons/Differences = NR. Definition of CI: “Although it is subjective, many would agree that the clinical significance of a difference of one-quarter of a standard deviation between a study group and its controls would be considered minor; one-half of a standard deviation, moderate; and one standard deviation, substantial” (p.18). % CI = NR

Note:

*McInnes and colleagues identified these studies as showing that *all* subjects had cognitive impairment [1].

**McInnes et al. identified these studies as showing that *some* subjects had cognitive impairment.

˟McInnes et al. identified these studies as showing that *no* subjects had cognitive impairment. First author, year published, and country are listed. Comp MTBI = percentage who have macrostructural trauma-related intracranial abnormalities visible on computed tomography or magnetic resonance imaging. Ages and recruitment settings are provided for the MTBI and control groups. IQ = Whether the original study measured estimated pre-morbid intellectual functioning or current intellectual functioning with one or more tests. Psych. = Whether the original study explicitly assessed psychological symptoms, such as depression and anxiety. This does not necessarily mean that these variables were used in statistical models to control for their potential effect when assessing for cognitive differences between groups. Comparisons/Differences: Number of group comparisons for cognitive outcomes (i.e., the number of test scores that were analyzed/compared) and the number of statistically significant group differences. CI = Cognitive Impairment. % CI = the % classified as impaired if the original authors had a criterion for impairment, applied it to the sample, and reported the percentage. NR = not reported. McInnes and colleagues cited Xu et al. in their original article, but this article was a mouse study on MTBI. During the review process for this manuscript, the correct Xu et al. article was identified.

McInnes et al. [1] identified 12 studies, at the 3 month post-injury time period, that they thought revealed *all subjects* to have cognitive impairment [23, 34, 38, 39, 43–45, 51–55] (demarcated with an asterisk in Table 1), 4 studies that had both cognitively impaired and unimpaired participants [22, 24, 30, 59] and 4 studies that they did not think revealed cognitive impairment in any participants [33, 56–58]. The samples and research methods varied considerably across these studies (see Table 1). Only five studies [22–24, 30, 59] used a methodology in which individual subjects were classified as having cognitive impairment. None of the original authors of the studies stated or concluded that *all* subjects with MTBIs were cognitively impaired.

McInnes et al. [1] identified 6 studies, at the 6 month post-injury time period, that they thought revealed *all* subjects to have cognitive impairment [25, 34, 39, 41, 55, 60] (demarcated with an asterisk in Table 1), 5 studies that revealed cognitive impairment in some subjects [26, 27, 30, 46, 61] and 1 study that they did not think revealed cognitive impairment in any subjects [35]. For two of the studies, multiple statistical comparisons of test scores between the MTBI group and the control group were conducted, with only one statistically significant result [34, 41]. Only three studies [25–27] used a methodology in which individual subjects were classified as having cognitive impairment. The original authors of the studies did not state or conclude that *all* subjects with MTBIs were cognitively impaired.

McInnes et al. [1] identified 11 studies, at the 12 month post-injury time period, that they thought revealed all subjects to have cognitive impairment [28, 31, 39–41, 47, 55, 62–65] (demarcated with an asterisk in Table 1), 1 study that revealed cognitive impairment in some subjects [46, 61] and 7 studies that they did not think revealed cognitive impairment in any subjects [32, 33, 48–50, 58, 66]. None of the original authors of the 20 studies concluded that *all* subjects with MTBIs were cognitively impaired. Some of these studies conducted numerous statistical comparisons and identified only one or two significantly different test scores [28, 40, 41, 64]. One study that McInnes et al [1] reported all subjects had cognitive impairment actually found no statistically significant differences between the MTBI and control group at the 12-month follow-up [55], and another study cited by McInnes et al [1] as showing evidence of cognitive impairment did not actually present, analyze, or interpret any cognitive test scores [47]. Only four studies [28, 31, 32, 61] used some sort of methodology in which individual subjects were classified as having cognitive impairment. One study enrolled 69 patients with MTBIs but only 16 underwent neuropsychological testing at one year following injury [28]. In this study, the MTBI group had significantly lower scores on only 2 of 23 test scores. The definition of cognitive impairment in this subgroup tested one year following injury was having at least one score that was 1.5 SDs below the normative mean. In the MTBI group, 69% had at least one low score. However, 44% of the control group also had at least one low score.

McInnes et al. [1] identified 8 studies, at greater than one year post-injury time period, that they thought revealed all subjects to have cognitive impairment [40–42, 65, 67–69] (demarcated with an asterisk in Table 1), 1 study that revealed cognitive impairment in some subjects [29] and 1 study that they did not think revealed cognitive impairment [32]. Several studies conducted numerous statistical tests and reported only one or two significant findings (e.g., [41, 67–69]). Only three studies [29, 32, 65] used a methodology in which individual subjects were classified as having cognitive impairment. None of the original authors of the 10 studies concluded that *all* subjects with MTBIs were cognitively impaired.

## Discussion

### Low neuropsychological test scores may or may not reflect acquired cognitive impairment

When inferring the cause of low neuropsychological test scores, it is important to appreciate that a person might obtain a low score due to situational factors, such as a lapse of attention, temporary distraction, not fully understanding the instructions, or low enthusiasm or motivation for testing. Moreover, a substantial percentage of healthy people with no prior brain injuries will obtain one or more low test scores when administered a battery of cognitive tests [70–81]. Researchers repeatedly have shown that it is very common for children [82], adults [76, 83], and older adults [84], with no known clinical conditions that might affect cognition, to obtain at least one low score when a battery of tests measuring multiple cognitive domains is administered [81]. As the number of test scores increases, the probability of a healthy person obtaining one or more low scores increases [79, 85]. The probability of obtaining a low score varies based on the *a priori* cutoff for defining a low score. For example, some clinicians and researchers define a low score as greater than 1 standard deviation below the mean, 1.5 standard deviations below the mean, or 2 standard deviations below the mean. Obtaining at least one low test score is also common in healthy people who are administered several tests within a cognitive domain, such as working memory [83, 85], learning and memory [77, 78, 84], speed of processing [83, 85], and executive functioning [86, 87]. Using one study from this review as an example, Rieger et al. [23] classified patients based on whether or not they had a single below average score (i.e., one cognitive test score < 30^th^ percentile). They reported that 96% of the MTBI group met this threshold, but so did 85% of their orthopedic control patients.

Demographic and personal characteristics also are associated with the probability of obtaining low cognitive test scores. African Americans and Hispanics, on average, obtain more low scores than Caucasians [88–93]. Level of education is associated with test performance; those with terminal high school diplomas obtain more low scores than those with university degrees [94]. Moreover, intelligence is correlated with neuropsychological test performance, so those with below average intelligence will obtain more low scores than those with average intelligence [78, 81, 94–99]. Therefore, low neuropsychological test scores may or may not reflect cognitive impairment following MTBI in individual cases. Per Tables 3–6 in the McInnes et al. review [1], 26.7% (n = 12/45) of the studies attempted to match MTBI patients to controls based on socioeconomic status, 57.8% (n = 26/45) matched for education, and 11.1% (n = 5/45) matched for race. Based on our review of these studies, fewer than half assessed intellectual functioning (see Table 1; n = 21/44 studies; 47.7%). Further, many of the studies that measured intelligence used it as an outcome variable that they thought may have been affected by MTBI. Most of these studies did not match for it between groups, control for it in statistical analyses, or discuss it as a potential confound when interpreting their results [e.g., [65]]. Current mental health problems, such as depression or anxiety, can influence cognitive test scores in patients without a MTBI [100] and in patients following a MTBI [101]. Of the studies in this review, 47.7% (n = 21/44) assessed for emotional symptoms (see Table 1), with very few of these studies accounting for the effect of emotional symptoms in their analyses or their interpretation of cognitive test results.

## Conclusions

Cognitive impairment can occur following a TBI of any severity. Even very mild TBIs at least temporarily impact cognition [102]. The risk of persistent or permanent cognitive impairment increases in association with the severity of the brain injury [12, 14, 103–106]. There is a considerable risk for long-term cognitive deficits after a moderate or severe TBI [14, 103, 107], though the type and severity of residual cognitive deficits is variable. Following a MTBI, cognitive impairment, as measured by neuropsychological tests, is likely to improve and resolve in the initial days, weeks, or months [2, 12]. Patients with structural abnormalities on computed tomography or magnetic resonance imaging, referred to as having a complicated MTBI, tend to perform somewhat more poorly on neuropsychological tests than patients with uncomplicated MTBIs in the first two months following injury [108–112]. However, sustaining a complicated MTBI may not increase the risk of long-term (i.e., >6 months) cognitive deficits [113, 114].

The running header for the scoping review by McInnes and colleagues [1] asserts “A single mTBI chronically impairs cognitive function.” The article has the potential to misinform scientists, clinicians, and the public. Some recently published articles have cited the McInnes scoping review as illustrating a high rate of cognitive impairment following “concussion” or MTBI [115–118]. Clinicians who review and accept the findings of McInnes et al. will be misinformed and potentially communicate an inaccurate prognosis to patients with a single MTBI. Some patients may personally misinterpret the literature as suggesting they will suffer long-term cognitive deficits following a single MTBI through their own review of this open access article.

We believe that the review by McInnes et al., especially when taken together with the aggregated literature over the past 50 years, does *not* support the conclusion that approximately half of individuals who sustain a single mild traumatic brain injury (MTBI) experience long-term cognitive impairment. Their scoping review included quantitative analyses based on flawed methodology. Moreover, they did not assess the quality of included studies or exclude studies with a high risk of bias. We re-reviewed the articles they identified and found that the articles themselves do not support their conclusions. Their scoping review reached conclusions that are discrepant from several prior systematic reviews involving meta-analysis [2–15], which consistently reached the conclusion that the impact of MTBI on neuropsychological performance becomes undetectable at the group-level by three months post injury. These prior systematic reviews do not provide an estimate of the incidence of chronic cognitive impairment following MTBI (i.e., risk of long-term deficits in an individual patient), but suggest that it is low [2].
